# Hypoxia Alters Cell Cycle Regulatory Protein Expression and Induces Premature Maturation of Oligodendrocyte Precursor Cells

**DOI:** 10.1371/journal.pone.0004739

**Published:** 2009-03-09

**Authors:** Ravi Shankar Akundi, Scott A. Rivkees

**Affiliations:** Yale Child Health Research Center, Section of Developmental Biology & Endocrinology, Yale University School of Medicine, New Haven, Connecticut, United States of America; Emory University, United States of America

## Abstract

**Background:**

Periventricular white matter injury (PWMI) is a common form of brain injury sustained by preterm infants. A major factor that predisposes to PWMI is hypoxia. Because oligodendrocytes (OLs) are responsible for myelination of axons, abnormal OL development or function may affect brain myelination. At present our understanding of the influences of hypoxia on OL development is limited. To examine isolated effects of hypoxia on OLs, we examined the influences of hypoxia on OL development *in vitro*.

**Methodology/Findings:**

Cultures of oligodendrocyte precursor cells (OPCs) were prepared from mixed glial cultures and were 99% pure. OPCs were maintained at 21% O_2_ or hypoxia (1% or 4% O_2_) for up to 7 days. We observed that 1% O_2_ lead to an increase in the proportion of myelin basic protein (MBP)-positive OLs after 1 week in culture, and a decrease in the proportion of platelet-derived growth factor receptor α (PDGFRα)-positive cells suggesting premature OL maturation. Increased expression of the cell cycle regulatory proteins p27^Kip1^ and phospho-cdc2, which play a role in OL differentiation, was seen as well.

**Conclusions:**

These results show that hypoxia interferes with the normal process of OL differentiation by inducing premature OPC maturation.

## Introduction

Periventricular white matter injury (PWMI) is one of the most common forms of brain injury in premature infants and is characterized by diffuse hypomyelination [1]. Risk factors for PWMI include hypoxia and poor cerebral blood flow [2], [3]. Although the cause of PWMI is not clear, significant evidence suggests that abnormal oligodendrocyte (OL) lineage development plays a role in PWMI causation [4].

OLs are responsible for myelination of the central nervous system [5]. OLs are generated in the neuroectodermal cells of the subventricular zone and migrate, proliferate, and differentiate into mature myelin synthesizing cells [6]. Four stages of OL differentiation are recognized [7], including oligodendrocyte precursor cells (OPCs), late OL progenitors, immature OLs, and mature OLs.

Early OPCs express the A2B5 antigen and the platelet-derived growth factor receptor alpha (PDGFRα) [8]. Late OL progenitors express NG2 and the O4 antigen [9]. These cells are mitotically active and migratory. Immature OLs express the galactocerebroside (GalC), O1 and O4 antigens [10]. Immature OLs are postmitotic cells and do not migrate. These cells differentiate into mature OLs that express myelin specific proteins, including myelin basic protein (MBP) [5].

OL development has been extensively studied in vitro. OL development is influenced by growth factors (GFs) that include basic fibroblast growth factor (bFGF) and platelet-derived growth factor (PDGF) [11], [12]. In the absence of these factors, OLs cease to proliferate and undergo differentiation [13], [14].

In general, cellular maturation is influenced by cell-cycle proteins [15], [16]. Progression from G_1_ to S phase is regulated by the retinoblastoma protein (Rb) [15]. Phosphorylation of Rb by cyclin-dependent kinases (CDKs) leads to the transcription of S-phase genes [17]. The onset of mitosis (G_2_ to M transition) requires the activation of cdc2 kinase [18]. Activation of cdc2 allows the phosphorylation of downstream substrates required for mitosis [19].

In OLs, cell-cycle proteins also influence cell maturation[16]. Negative regulators of CDKs exist, and elevated levels of CDK inhibitors induce cells to exit the cell cycle by arresting G_1_ to S transition and preventing the onset of mitosis [20]. Levels of p27 progressively rise as OPCs differentiate into mature OLs [21].

Currently our understanding of the effects on hypoxia on the developing brain is at early stages, and we know little about how hypoxia contributes to myelination defects during development. We hypothesized that hypoxia may alter OL cell lineage development contributing to PWMI. To address this issue we examined effects of hypoxia in isolated OLs. We now report that hypoxia leads to surprising alterations in OPC maturation by inducing premature maturation.

## Materials and Methods

### Materials

Cell culture materials were obtained from Invitrogen (Carlsbad, CA). Basic fibroblast growth factor (bFGF) and platelet-derived growth factor (PDGF) were obtained from Roche (Indianapolis, IN). Tissue culture plates, including poly-D-lysine (PDL)-coated flasks or coverslips, were from BD Biosciences (San Jose, CA). Antibodies against PDGF receptor α (PDGFRα), phospho-cdc-2, phospho-Rb and the ribosomal S6 protein were from Cell Signaling Technologies (Beverley, MA), anti-CNP, anti-GalC and anti-MBP antibodies were from Covance BabCo (Berkeley, CA), anti-p27^Kip1^ from Santa Cruz Biotechnology (Santa Cruz, CA), anti-Hif-1α from Cayman Chemicals (Ann Arbor, MI) and anti-β-actin from Sigma (St. Louis, MO). HRP-conjugated secondary antibodies were from Santa Cruz Biotechnology (Santa Cruz, CA). Alexa Fluor 488 and Alexa Fluor 530-conjugated secondary antibodies were from Invitrogen (Carlsbad, CA). All other chemicals were from Sigma Chemical Co.

### Cell culture and reagents

OLs were cultured as described [22]. Primary mixed glial cultures were obtained from postnatal day 1 rat brains, and cells were grown in poly-D-lysine (PDL)-coated T75 flasks in Dulbecco's modified Eagle's medium (DMEM) containing 100 U/ml penicillin, 100 µg/ml streptomycin, 0.25 µg/ml amphotericin B and 10% heat-inactivated FCS (Invitrogen). Cells were incubated at 37°C in 5% CO_2_, and media was changed every 2–3 days until cells reached 80% confluence (8–9 days). Microglia, which loosely attach on top of the mixed glial cultures, were removed on day 8 by mechanical shaking at 200 rpm for 1 hr at 37°C. Fresh media was added to the cultures, which were shaken overnight at 250 rpm to isolate oligodendrocyte precursor cells (OPCs). Floating cells were collected and plated in T75 flasks. After 30 min, loosely attached OPCs were isolated and replated on PDL-coated plates. This method has shown to yield cultures with 99% purity of OPCs in our hands. We also assessed the purity of our cultures by staining with specific markers such as CD11b, which label microglia, and glial fibrillary acidic protein (GFAP), that labels astrocytes [23], [24]. We did not find any staining for GFAP, and less than 1% cells stained for CD-11b. More than 98% cells stained for NG2 chondroitin sulphate proteoglycan, suggesting a pure population of OPCs.

OPCs were plated at a density of 3×10^4^ cells/cm^2^ on PDL-coated dishes for Western blotting and fluorescence-activated cell sorting (FACS) analysis, and on PDL-coated 12-mm glass coverslips at a density of 7.5×10^4^ cells/coverslip for immunostaining and ELISA. Cells were left overnight in DMEM containing antibiotics and 10% FCS. The following day, medium was replaced with fresh DMEM containing growth factor (GF) mix (10 ng/ml each of bFGF and PDGF) and N-2 supplements (100 µg/ml transferrin, 5 µg/ml insulin, 6.3 ng/ml progesterone, 16.1 µg/ml putrescine and 5.2 ng/ml selenite), in addition to antibiotics and 0.5% heat-inactivated FCS.

All experiments were performed the following day (*in vitro* day 2). Media was changed and cells were treated with fresh media, in the presence or absence of GFs. Hypoxia was maintained by incubating the cells in a Napco 7001 incubator (Precision Scientific, Chicago), which contained a humidifying water pan, and infused with 5% CO_2_ and 95% N_2_ to achieve the desired concentration of hypoxia. Cells were removed briefly every day to add GFs in the treatment groups. Media was changed on alternate days.

### BrdU and CuQuant

The proliferation of cells was measured by detecting the incorporation of the thymidine analog bromodeoxyuridine (BrdU) as described [11], [25]. BrdU (10 µM) was added for 12 h before the study was terminated.

The CyQUANT cell proliferation assay kit (Molecular Probes, Eugene OR), which measures the total nucleic acid content [26], was used according to manufacturer's recommendations.

### Immunocytochemistry

To detect intracellular hypoxia, 0.1 mM pimonidazole (Chemicon, Temecula, CA) was added to cells 2 hrs before fixation in 4% PFA. Pimonidazole forms adducts in cells in which the oxygen concentration is less than 14 µM [27]. These adducts were detected using an antibody supplied with the kit.

Morphology of OLs was assessed by staining with actin-specific Alexa Fluor 488-conjugated-phalloidin (50 U/ml) on paraformaldehyde (PFA)-fixed cells [28]. Cells were observed using fluorescent microscopy.

Stage-specific maturation of OLs was assessed on 4% PFA-fixed cells using rabbit anti-PDGFRα, rabbit anti-GalC, and mouse anti-MBP (at 1∶500 or 1∶1000 dilution). Cells were permeabilized with 90% methanol for 30 min on ice, and blocked in 5% goat serum containing 0.1% Triton X-100 for 2 hrs. Double labeling was done by adding rabbit anti-PDGFRα and mouse anti-MBP (both at 1∶1000 dilution, incubated overnight at 4°C) and visualized using Alexa 488-conjugated goat anti-rabbit or Alexa 546-conjugated goat anti-mouse antisera, respectively. Cells were counterstained with DAPI to stain nuclei, and were observed under a fluorescent microscope. The percentage of stages-specific OLs was determined as the number of labeled cells divided by the number of DAPI-stained nuclei.

### Western blot analysis

Cells were washed in ice-cold phosphate buffered saline (PBS) and lysed using hot lysis buffer containing 42 mM tris-HCl (pH 6.8), 1.3% sodium dodecylsulfate (SDS), 6.5% glycerol, and 0.1 mM sodium orthovanadate. Protein concentrations were determined using the bicinchoninic acid method (BCA kit, Pierce Technologies, Rockford, IL). Before loading, samples were mixed with 10 mM dithiothreitol and 0.1% bromophenol blue, and boiled for 5 min. Proteins were separated on an SDS-polyacrylamide gel (7.5% for pRb and Hif-1α and 12% for MBP, cdc-2 and p27), transferred to a polyvinylidene fluoride (PVDF) membrane, and blocked with 5% non-fat dry milk in 20 mM Tris-HCl, pH 7.6, 150 mM sodium chloride and 0.1% tween-20 (TBS-T) for 1 hr. Primary antibodies were diluted in blocking buffer (1∶200 for p27^Kip1^ and Hif-1α, and 1∶1000 for all other antibodies), and incubated overnight at 4°C with gentle shaking. The antibody against MBP detects all isoforms of MBP (17–22 kDa) except the 14 kDa isoform. The antibody against cdc-2 detects cdc-2 when phosphorylated at Tyr 15. The antibody against retinoblastoma (Rb) detects Rb when phosphorylated at Ser 807. Secondary antibodies were diluted in blocking buffer and incubated for 1 hr at RT. HRP-conjugated goat anti-rabbit antibody (1∶5000 dilution) was used in all cases, except for MBP, CNP and β-actin for which HRP-conjugated goat anti-mouse (1∶5000) was used. After washing, proteins were detected using an enhanced chemiluminescence kit (Pierce Biotech).

### Enzyme Immunoassay

Cells were cultured in PDL-coated 24-well plates, and media was collected at 3 hrs, 6 hrs, 12 hrs, 24 hrs, 48 hrs and 96 hrs of culture. Levels of vascular endothelial growth factor (VEGF A) were measured using a commercially available kit (R&D Systems, Minneapolis, MN). The kit recognizes both the 164 and 120 amino acid residue forms of mouse VEGF A.

### Statistical analysis

All experiments were performed in triplicate with cells obtained from different litters. Data are represented as mean±SEM. Comparisons among multiple groups were performed by ANOVA or by Student's *t*-test for two-group comparisons. Differences were considered significant at *p*<0.05.

## Results

To examine the effects of hypoxia on OLs, without the potential confounding effect of other cell types, studies were performed on primary OL cultures. OL differentiation was examined at standard cell culture conditions (21% O_2_) and at two lower concentrations of oxygen (4% and 1% O_2_). In initial experiments both 1% and 4% O_2_ concentrations were used. When it became apparent that 1% O_2_ did not result in cell death, in several studies, 1% oxygen alone was used to maximize hypoxic effects.

**Figure 1:**
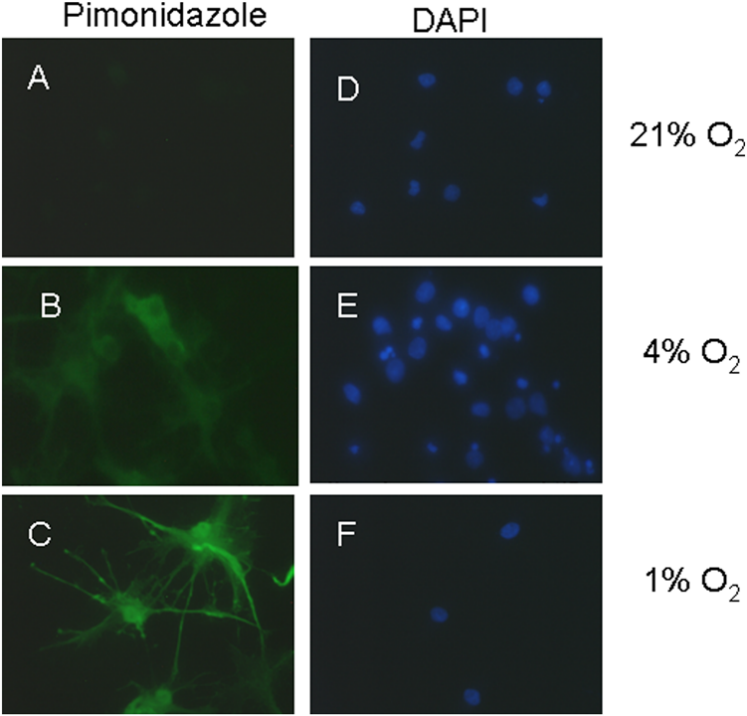
Demonstration of intracellular hypoxia. Intracellular hypoxia was assessed by incubating cells with pimonidazole (A, C and E), which forms adducts at pO_2_ of <10 mm Hg (green). Cells are counterstained with DAPI (B, D, and F; blue). Note absent staining in 21% O_2_ (A), moderate staining in 4% O_2_ (C), and high level staining in 1% O_2_ (E). Scale bars = 10 um. Data shown are representative of four separate studies.

To assess that cells were hypoxic at the lower concentrations of oxygen used, we used pimonidazole which binds to cells that have oxygen concentrations less than a pO_2_ of 10 mm Hg at 37°C. Pimonidazole adducts could be observed in cultures maintained at 1% O_2_, but not in cultures at 21% O_2_ (Fig. 1). At 4% O_2_, pimonidazole labeling was observed, but at lower intensity than at 1% O_2_.

**Figure 2:**
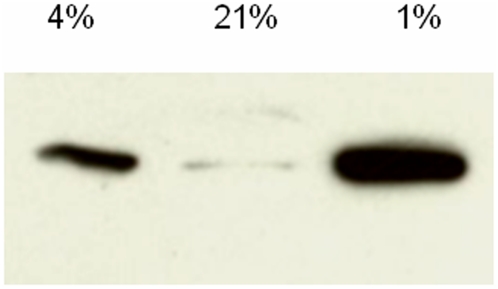
Hypoxia influences on Hif-1α. Whole cell lysates of OLs cultured in 21%, 4% or 1% O_2_ for 6 hrs. Cell lysates were immunoblotted for Hif-1α (120 kDa). 50 ug of protein per lane. Data shown are representative of three such studies.

To provide additional biochemical evidence that the conditions used induced hypoxia, we investigated if cultures maintained at 1% O_2_ showed an increase in hypoxia-inducible factor 1α (Hif-1α). In cultures maintained at 4% and 1% O_2_, increased Hif-1α expression was seen at 6 hrs (Fig. 2), above that seen in 21% O_2_ (P<0.01 for 4% and 1% O_2_ vs. 21%; ANOVA). At 24 and 48 hrs of 1% O_2_ exposure, increased Hif-1α expression was not observed (not shown), which is consistent with studies in other systems showing that Hif-1α expression in hypoxia falls after hypoxia exposure onset[29], [30].

**Figure 3:**
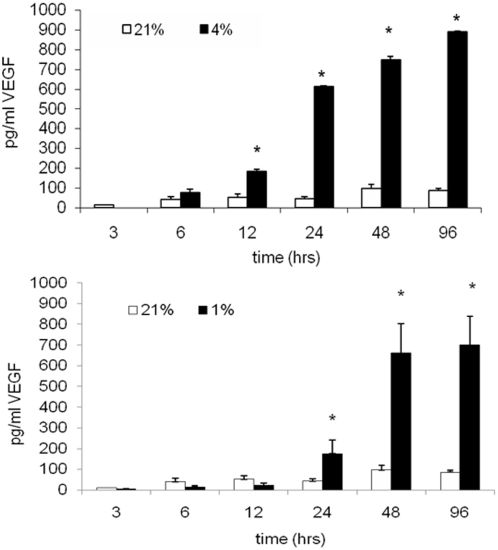
Hypoxia influences on VEGF A. OLs were cultured in the presence of GFs in 21%_2_, 4% or 1% O_2_. Cell culture media was collected at 48 hrs, 96 hrs, and 7 days and levels of secreted VEGF A were measured by ELISA (*p<0.01; ANOVA vs. 21% O_2_). Data shown are representative of three separate studies.

We also investigated if there was an increase in levels of Hif-1α-regulated synthesis and release of vascular endothelial growth factor A (VEGF A). For these studies, cells were cultured in 21%, 4% and 1% O_2_, in the presence or absence of GFs. VEGF A concentration in the media was assessed after 1, 4, and 7 days. We observed that 4% and 1% O_2_ conditions resulted in increased VEGF A levels, as compared to 21% O_2_ (Fig 3). Interestingly, at 12 and 24 hrs, VEGF A levels were greater at 4% than 1% O_2_ (Fig. 3). Yet, at 96 hrs and 7 days, there no differences in VEGF A levels were observed between 4% and 1% O_2_. (p>0.05, ANOVA; Fig. 3). These data show that cultures maintained at low concentrations of oxygen show sustained VEGF A release.

After validating that the OLs were hypoxic under the conditions used, we assessed effects on OL survival and maturation. OL proliferation is influenced by growth factors (GFs) PDGF and FGF, and deprivation of these factors leads to OL maturation *in vitro* [31]. To examine effects of hypoxia on OL development, cells were studied in the presence or absence of GFs.

**Figure 4:**
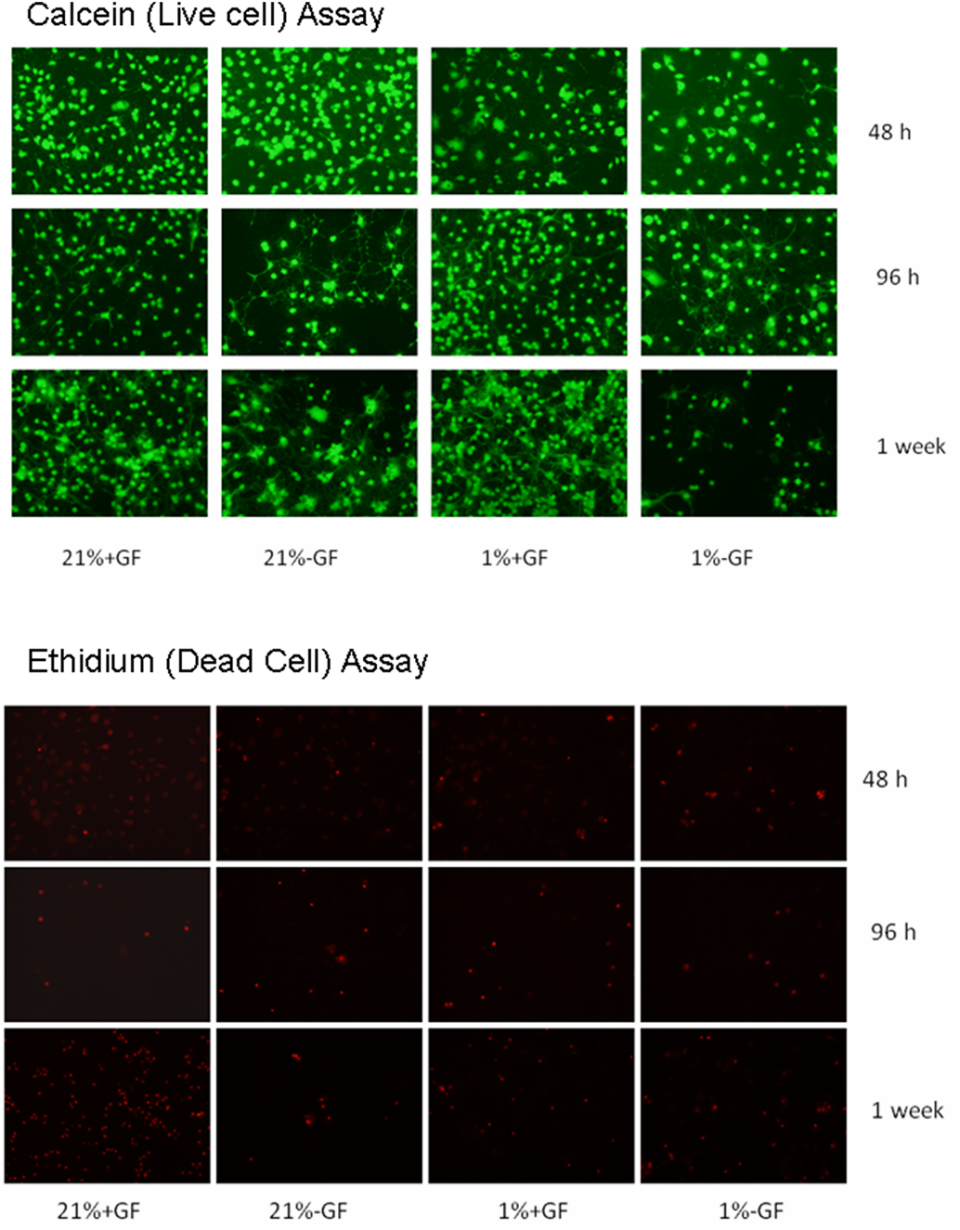
Hypoxia influences on cell viability. Images of calcein (live) and ethidium (dead) cells labeled OLs cultured in the presence or absence of GFs in 21%_2_, 4% or 1% O_2_ for 48 hrs, 96 hrs, and 7 days. Images shown are representative of 4 separate studies.

**Figure 5:**
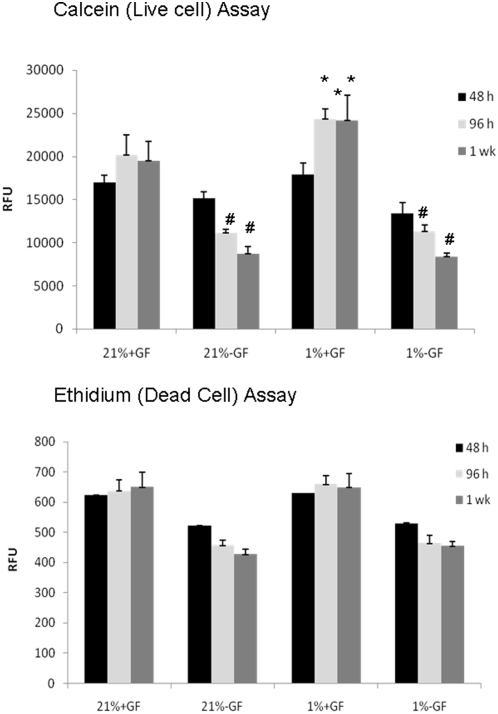
Hypoxia influences on cell viability. Calcein (live) and ethidium (dead) assays of OLs cultured in the presence or absence of GFs in 21%_2_, 4% or 1% O_2_ for 48 hrs, 96 hrs, and 7 days. Relative florescence units are shown (RFU). Data are averages of 4 separate studies. (*p<0.01; ANOVA vs. 21% O_2_+GF; #, P<0.05 ANOVA vs. 21% O_2_+GF).

We first examined if hypoxia induced cell death at either 21% or 1% O_2_ in the presence or absence of GFs using calcein (4 µM) that labels live cells, and ethidium homodimer (4 µM) that is taken up by dead cells [32]. Assays were preformed at 48 hrs, 96 hrs, and one week in culture (Figs. 4 and 5). At 48 hrs, calcein labeling was similar in the presence or absence of GFs (p>0.05; ANOVA; Fig. 4). At 96 hrs, calcein labeling was greater in the presence of GFs in 1% than 21% O_2_ (p<0.05; ANOVA; Fig. 5). At 7 days, calcein labeling was greater in the presence of GFs in 1% than 21% O_2_ (p<0.05; ANOVA; Figs. 4 and 5).

At 48 hrs, ethidium labeling was similar in the presence or absence of GFs in 21% or 1% O_2_ (p>0.05; ANOVA; Fig. 5). At 96 hrs, ethidium labeling was similar in the presence or absence of GFs in 21% or 1% O_2_ (p>0.05; ANOVA; Fig. 5). At 7 days, ethidium labeling was similar in the presence or absence of GFs in 21% or 1% O_2_ (p>0.05; ANOVA; Figs. 4 and 5).

Next TUNEL assays were performed. There were slightly less dead cells in 1% than 21% O_2_. In the 21% O_2_, 7.8±0.2% of cells were TUNEL-positive in cultures maintained at one week, whereas about 5.6±0.2% of cells were TUNEL-positive following being cultured in 1% O_2_ for one week (p<0.05; t-test).

Cell numbers were also examined at the end of the one week culture period. At the end of one week, there were 27±4% more cells (p<0.01; t-test) in 1% O_2_ compared with 21% O_2_ in GF-treated cultures. These observations are consistent with the calcein data showing more live cells at 7 days in 1% O_2_ compared with 21% O_2_ in GF-treated cultures. No significant difference in cell number was seen between 1% and 21% O_2_ in GF-deprived cultures.

**Figure 6:**
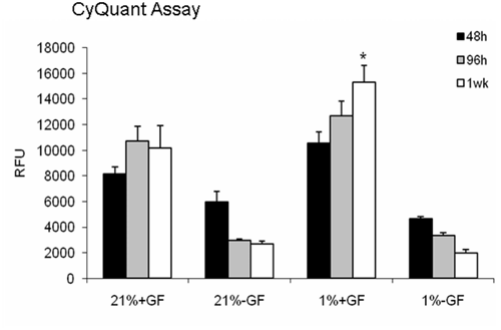
Hypoxia influences on cell number. CyQuant analysis was performed at 48 hrs, 96 hrs, and at 7 days for OLs cultured in the presence or absence of GFs in 21%_2_ or 1% O_2_. Relative florescence units are shown (RFU). In GF-treated cultures in 1% O_2_, levels was similar to the 21% O_2_ group at 46 and 96 hours (p>0.05; ANOVA). At 7 days, levels were greatest in the cells cultures in 1% O_2_ and GFs (*, p<0.05; ANOVA). Data are averages of three separate studies.

Cell proliferation was assessed using BrdU at the end of one week in GF-treated culture. No significant differences in numbers of labeled cells between 1% and 21% O_2_ were observed (p>0.05). As a complementary approach, CyQuant analysis was performed at 48 hrs, 96 hrs, and at one week (Fig. 6). In GF-treated cultures in 1% O_2_, this cell proliferation index was similar to the 21% O_2_ group at 46 and 96 hours (p<0.05; ANOVA). At one week, CyQuant labeling was greatest in the cells cultures in 1% O_2_ and GFs (p<0.05; ANOVA; Fig. 6).

**Figure 7:**
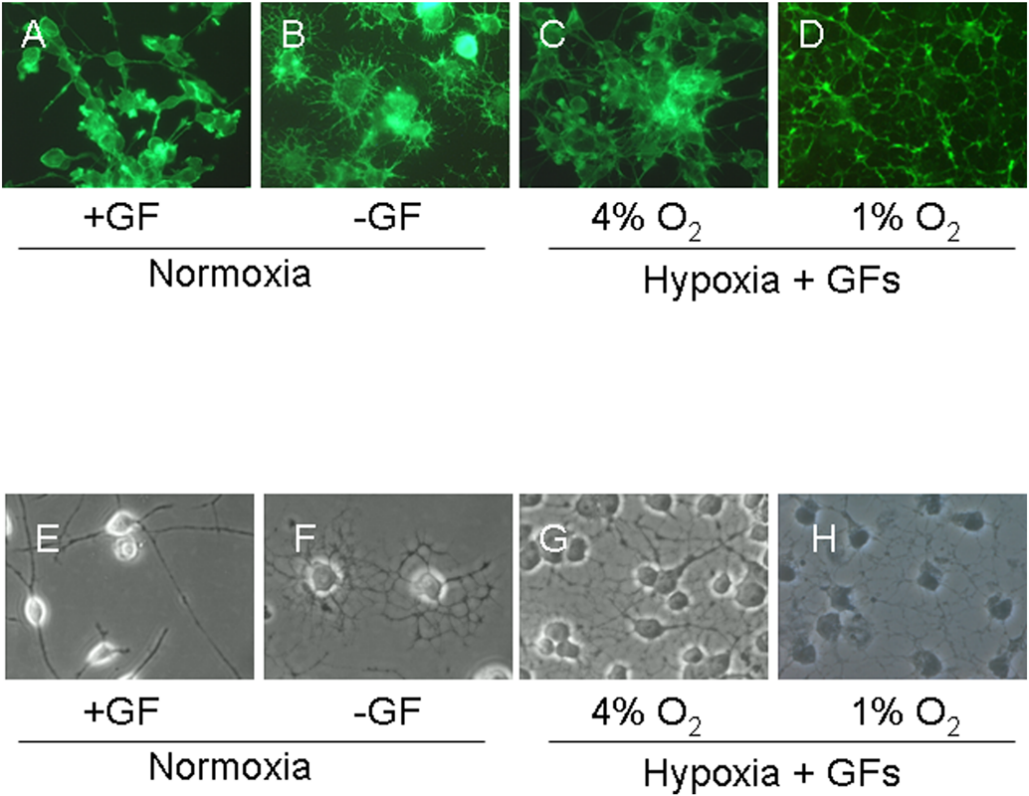
Changes in OL morphology in hypoxia. OLs were cultured in 21% O_2_ in the presence of GFs (A, E), in the absence of GFs (B, F), or under with GFs in 4% O_2_ (C, G) or 1% O_2_ (D, H) for 1 week. Fluorescence images of cells stained with actin-specific phalloidin (A–D) and phase-contrast images (E–H) of OLs show that cultures deprived of GFs (B, F) or cultures exposed to hypoxia (C, D, G, H) undergo morphological changes consistent with enhanced maturation. Scale bars = 10 um. Data shown are representative of five separate studies. At least ten separate coverslips were analyzed per study.

We next assessed influences of hypoxia on OL maturation by assessing changes in cell morphology using the actin filament-specific, phalloidin dye. Cells cultured in 21% O_2_ with GFs had small nuclei and few long processes (Fig. 7). GF deprivation resulted in shorter but extensive process, a characteristic of mature OLs. Cells cultured in 1% or 4% O_2_ in the presence of GFs showed more processes than the normoxic ones, although the processes were not as extensive as that seen normoxic, GF-deprived cultures (Fig. 7).

**Figure 8:**
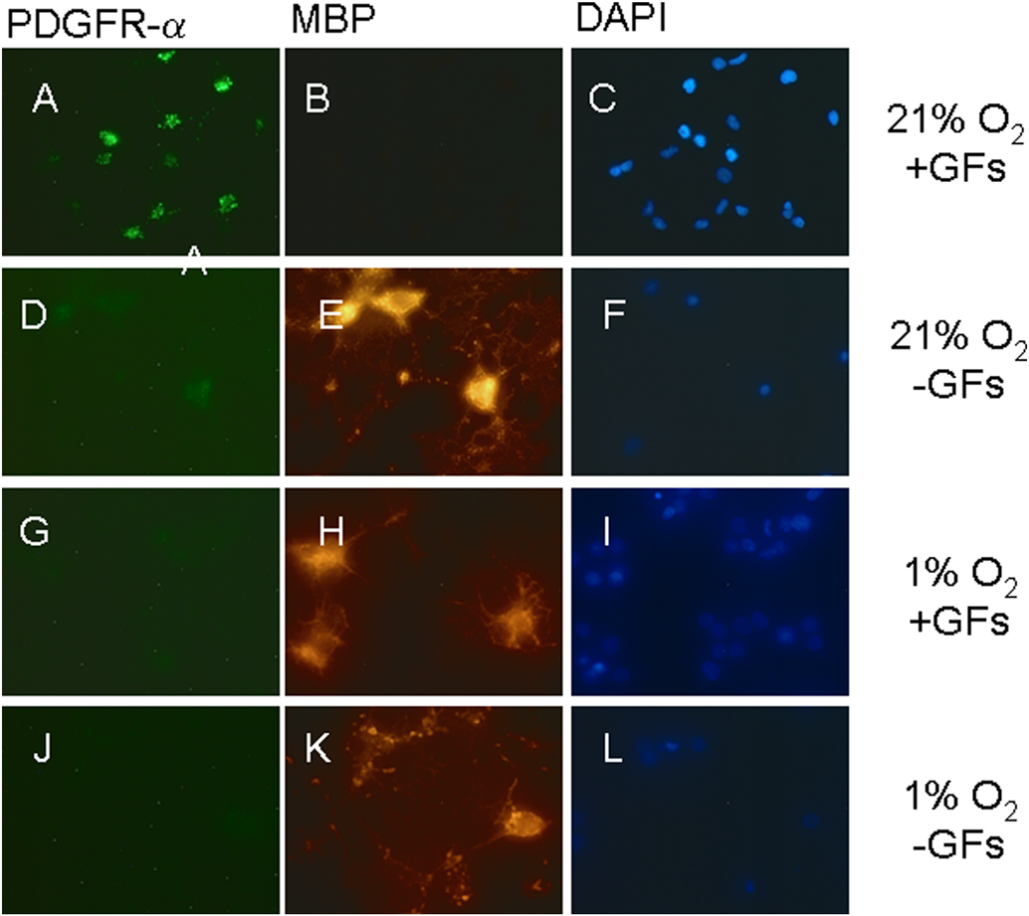
Hypoxia induces premature OL maturation. Images of OLs cultured for one week in 21% O_2_ in the presence (A–C) or absence (D–F) of GFs, or in 1% O_2_ with (G–I) or without (J–L) GFs. Cells were double-labeled for PDGFRα (A, D, G, J) or MBP (B, E, H, K) and counterstained with DAPI (C, F, I, L). A higher proportion of cells stained for PDGFRα (green) in 21% O_2_ whereas more MBP (red) labeled cells were observed in 1% O_2_ plus GF, or in GF-deprived cultures. OL differentiation was assessed from numbers of MBP-positive cells relative to total cell number (DAPI-positive nuclei). (* *p*<0.001 in comparison to cultures treated with GFs and maintained at 21% O_2_). Scale bars = 10 µm. Data shown are representative of three separate studies. At least ten separate coverslips were analyzed per study.

**Figure 9:**
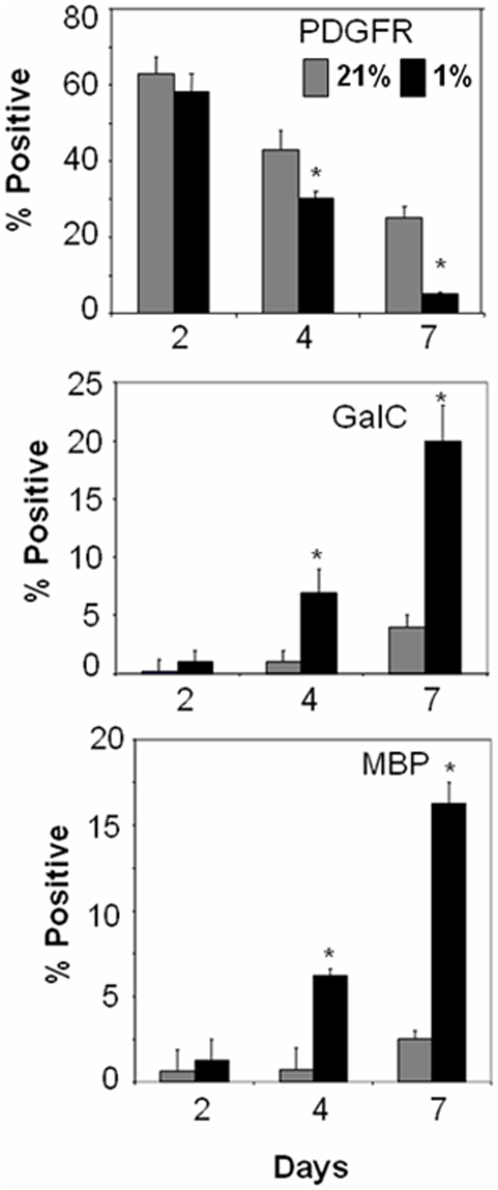
Hypoxia induces premature OL maturation. Percentages of cells that are positive for PDGFRα, GalC, or MBP cultured in 21% or 1% O_2_ plus GFs at 48 hrs, 96 hrs or 7 days in culture. * *p*<0.05 1% vs. 21% O_2_ at each time. Data shown are representative of three separate studies. At least three separate coverslips were analyzed per study.

As an additional measure to examine OL maturation during hypoxia, immunocytochemistry was performed on cultures maintained in 21% or 1% O_2_ for 7 days in the presence of GFs (Figs. 8 and 9). In the absence of GFs in 21% O_2_, there was differentiaton of OLs, and at 7 days in culture 23.5±2.26% of cells were MBP-positive and 1.03±0.26% of cells were PDGFR-positive, vs. 3.5±36% and 67±8% at 48 hrs, respectively (P,0.05, ANOVA). In the absence of GFs in 1% O_2_, there was also differentiation of OLs. At 7 days in culture, 30.4±2.32% of cells were MBP-positive and 0.59±0.26% were PDGFR-positive (Fig. 8).

In the presence of GFs in 21% O_2_, OL maturation was arrested, as 2.13±0.4% of cells were MBP-positive at 7 days (Figs. 8 and 9). However, in 1% O_2_ we observed maturation of OLs (Figs. 8 and 9). At 96 hrs and 7 days, compared to 48 hrs, we observed more MBP- and Gal-labeled cells and fewer PDGFRα,-labeled cells in 1% O_2_ than 21% O_2_ (p<0.05, ANOVA; Fig 9). At both 96 hrs and 7 days, increases in the proportion of MBP- and GalC positive cells in 1% O_2_ were associated with progressive reciprocal decreases in the proportion of PDGFRα labeled cells (p<0.05, ANOVA; Figs 8 and 9).

**Figure 10:**
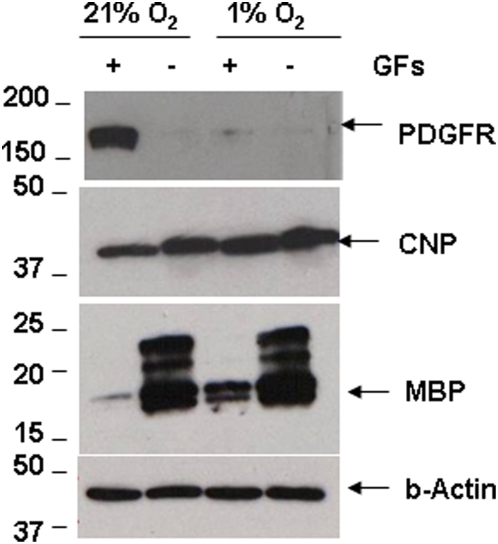
Hypoxia mediated acceleration of OL maturation. Cell lysates of OLs in 21% and 1% O_2_ for 1 week with GFs were analyzed by immunoblotting. Representative image shows decreased PDGFRα and increased MBP expression in 1% O_2_, and in cultures without GFs. β-actin loading control shown in bottom panel.

To further assess influences on OL maturation in hypoxia, cell lysates were tested for PDGFRα, CNP, and MBP expression by immunoblotting. OPCs were cultured in the presence or absence of GFs for one week in 21% or 1% O_2_. In 21% O_2_ with GFs, we observed high expression of PDGFRα and low MBP expression. PDGFRα expression was not seen in GF-deprived cultures or in cultures maintained at hypoxia (Fig. 10). In 1% O_2_ and GFs as we observed low PDGFRα levels and high MBP levels. The expression of CNP was also higher in 1% than 21% O_2_ in the presence and absence of GFs. as well. GF-deprived cultures in 21% O_2_ showed bands for most isoforms of MBP (17–22 kDa). In GF-treated cultures, only the lower band isoforms (17–19 kDa) of MBP were observed in 1% O_2_ (Fig. 10).

Because OLs exit the cell cycle to undergo differentiation [16], we examined cell-cycle regulatory kinases in cultures in different concentrations of oxygen. We examined three key proteins involved in cell cycle- cdc2, retinoblastoma (Rb) protein, and p27^Kip1^ [16]. For the analysis of cdc-2 and Rb, phosphorylation state-specific antibodies were used. The pRb antibody used is specific for phosphorylation site Serine 807/811 and does not recognize Rb phosphorylated at other sites such as Serine 608, Serine 780 or Serine 795.

**Figure 11:**
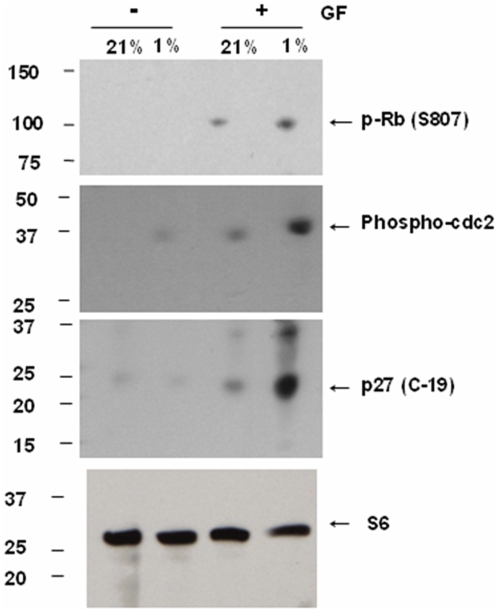
Changes in OL cell cycle proteins in 1% O_2_. Whole cell lysates from OLs cultured at 21% O_2_ or 1% O_2_ in the presence or absence of GFs, were analyzed by immunoblot analysis for phosphorylation state of cell cycle proteins Rb and cdc2, and for the negative regulator of cell cycle, p27^Kip1^. Ribosomal S6 protein was used as a loading control. The approximate sizes for Rb, cdc-2, p27^Kip1^ and ribosomal S6 protein are 110 kDa, 34 kDa, 27 kDa and 32 kDa, respectively. Representative Western blot from 3 different preparations shows an increase in phosphorylated forms of Rb and cdc-2, and total p27^Kip1^ levels in hypoxia. Data shown are representative of three separate studies.

**Figure 12:**
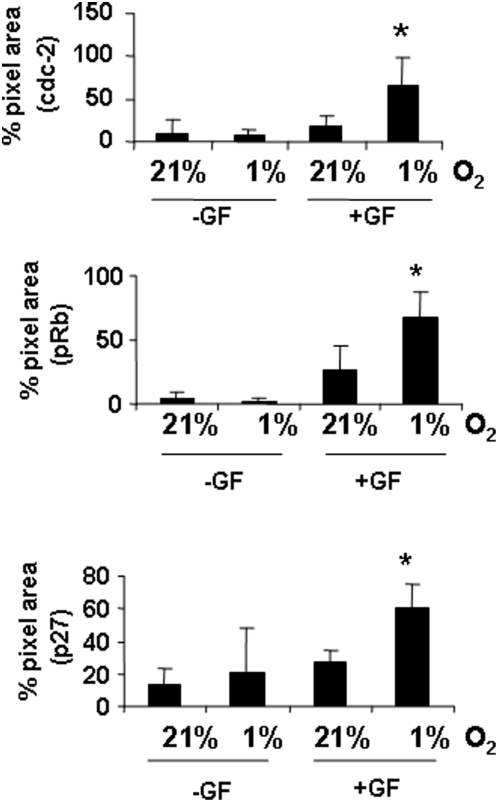
Changes in OL cell cycle proteins in 1% O_2_. Densitometric analysis of data in Figure 9 was done by normalizing protein levels to ribosomal S6 protein levels. The percent increase in the expression of cell cycle proteins is shown relative to levels in 21% O_2_ (* *p*<0.05, ** *p*<0.01). Data shown are representative of three separate studies.

**Figure 13:**
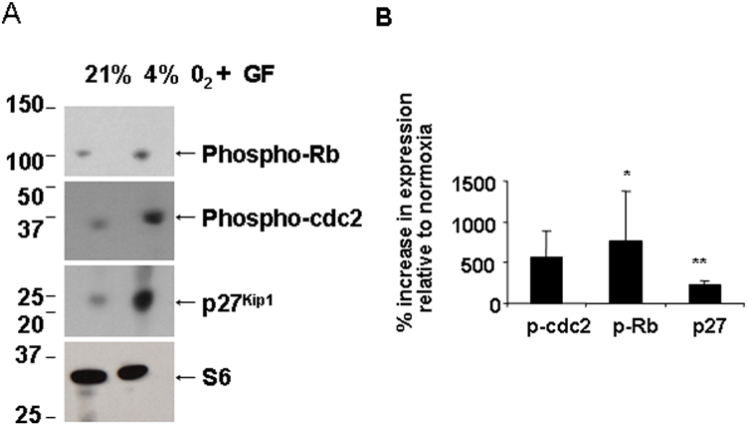
Changes in OL cell cycle proteins in 4% O_2_. Whole cell lysates from OLs cultured at 21% O_2_ or 4% O_2_ in the presence of GFs, were analyzed by immunoblot analysis for phosphorylation state of cell cycle proteins Rb and cdc2, and for the negative regulator of cell cycle, p27^Kip1^. Ribosomal S6 protein was used as a loading control. The approximate sizes for Rb, cdc-2, p27^Kip1^ and ribosomal S6 protein are 110 kDa, 34 kDa, 27 kDa and 32 kDa, respectively. Representative Western blot from 3 different preparations shows an increase in phosphorylated forms of Rb and cdc-2, and total p27^Kip1^ levels in hypoxia. (B) Densitometric analysis was done by normalizing protein levels to ribosomal S6 protein levels. The percent increase in the expression of cell cycle proteins is shown relative to levels in 21% O_2_ (* *p*<0.05, ** *p*<0.01). Data shown are representative of three separate studies.

Using Western blotting, we observed an increase in the phosphorylated form of cdc2 in GF-treated cultures, with higher expression seen in 1% O_2_ with GFs (Figs. 11 and 12). We observed a significant increase in the phosphorylated form of Rb in 1% O_2_ cultures treated with GFs (*p*<0.05). Increased expression of the negative cell cycle regulator p27^Kip1^ was observed in hypoxia-treated cells despite the presence of GFs (Figs. 11 and 12, *p*<0.01; ANOVA). In 4% O_2_ in the presence of GFs, we also observed increases in phosphorylated forms of cdc3, Rb and p27^Kip1^ (Fig. 13).

## Discussion

Abnormal myelination is a characteristic of PWMI in premature infants and can be induced by hypoxia alone in animal models of the condition [33]. The mechanisms by which hypoxia contributes to hypomyelination by influencing OLs is not known. Our findings suggest that hypoxia induces changes in OL cell cycle proteins to favor premature OL differentiation.

Hif-1α is involved in the transcriptional regulation of several genes including those encoding for VEGF [34]. We observed that 1% and 4% O_2_ triggered large increases in Hif-1α expression. We also found potent increases in the levels of VEGF A in 1% or 4% O_2_. Collectively these data show that OLs were hypoxic under the conditions used.

The growth factors bFGF and PDGF maintain OLs in a state of continued proliferation and inhibit differentiation [11]. Despite the presence of GFs, we observed OL maturation in hypoxia, as significant increases in the proportion of GalC and MBP-positive cells were seen after exposure to 1% O_2_ along with corresponding decreases in the numbers of PDGFRα-positive cells were seen in hypoxic cultures, showing a decrease in the proportion immature OL forms in hypoxia. It is important to note that used the conditions used, the total number of PDGFRα, GalC and MBP-labeled cells did not total 100%. It is possible that this reflects incomplete staining of OL cell types, as based on our previous studies, we have found that OLs cultures are >98% pure OLs when under the culture conditions used[25], [35]. Thus, even in conditions that normally maintain OL proliferation and inhibit differentiation, hypoxia can override normal anti-differentiation signals and trigger OL maturation.

It was interesting that more OLs were present when cultured in hypoxia than in room air using two different approaches. The cellular basis of this observation is not clear. Yet, since we observe more mature forms of OLs in hypoxia than normoxia, it is possible that mature OLs are more tolerant of hypoxia than immature forms.

Maturation of OPCs into mature OLs requires cells to exit the cell cycle [16]. We found that hypoxia induced p27^Kip1^ expression in OL cultures. P27^Kip1^ arrests cells in G_1_ phase, and allows cells to exit cell cycle and undergo differentiation [36], [37], [38]. In addition, we found higher levels of phosphorylated cdc2 in hypoxia-treated cultures. When phosphorylated, cdc2 remains inactive, thus substrates required for mitosis (M phase) are not active [19]. Thus, hypoxia leads to changes in the expression of cell cycle proteins in a manner that favors OL differentiation.

Based on our observations, we postulate that hypoxia-mediated premature maturation of OL cells will lead to a reduction in the pool of proliferating OPCs, possibly leading to a reduction in the total number of myelinating cells in the brain. These observations are consistent with our *in vivo* findings of a reduction in the proportion of Olig-2-positive cells the brains of animals reared in hypoxia and reduced myelination in hypoxia-reared mice [33]. In hypoxic cultures, PDGFRα-positive cells were observed to have processes and simplified arborization, similar to that observed in the brains of hypoxic mice [33]. In future studies, it will thus be important to quantify OL stage-specific subtypes in the brains of developing animals exposed to hypoxia to assess if our in vitro observations of premature OL maturation are also seen in vivo.

Overall, we find that hypoxia induces premature maturation of OPCs. We postulate that premature maturation of OPCs will lead to decreases in numbers of replicating OLs, leading to fewer myelinating OLs in the brain. As such, premature OPC maturation may contribute to hypomyelination in the developing hypoxic brain.
